# Vehicle Safety Post-Decade of Action for Road Safety: The Way Forward 

**Published:** 2019-08

**Authors:** Mehdi Shafieian, Dinesh Mohan

**Affiliations:** ^*a*^Amirkabir University of Technology (Tehran Polythenic), Iran.; ^*b*^Indian Institute of Technology Delhi, India.

## Abstract

As the “Decade of Action for Road Safety” reaches its final stages, the quest to look out for the next steps forward continues. The experiences from the Decade of Action will help us to define an effective roadmap for the next decade.

Vehicle safety has been one the areas that has been subjected to significant changes over the past decade with advancements in technology, especially in the field of active safety. This change in the field has been more tangible in High Income Countries (HICs). This note suggests a path to determine the effect of vehicle safety in Iran as a Low/Middle Income Country (LMIC), considering the road safety management structure of the country and introduces the priorities that need to be the subject of focus in further stages.

As the “Safe System Approach for Road Safety” focuses on reducing the fatalities and injuries, the interactions between human body and the surrounding area, the vehicle becomes an important issue. Therefore, vehicle designs that meet modern safety standards, with the goal of reducing the risk of occurrence of crashes and ensuing injuries become more prominent. As these advancements in active and passive safety features are being introduced in HICs, the nature of these technologies and limited resources in LMICs, require a deeper understanding of the effectiveness of these technologies would help us set more effective strategies in such countries.

A profound understanding of the causes of crashes and the following injuries, is an essential step in setting precise strategies to continue reducing fatalities. This issue has been one of the targets during the decade of action, but a structured regime with independence from involved key stakeholders, must be established to precisely determine the role and details of the involvement of vehicle design and technical aspects in occurrence of the crashes and following injuries.

As utilizing advanced technologies for upcoming future is a must to enhance the vehicle safety, considering the economic status of the country besides the evidences in the trend of crashes in Iran suggest effective interventions to achieve results in the shortest time, it would be important to examine the possibility of introducing some of the technologies in the shortest possible time, for example:

a. New Car Assessment Program (NCAP) ratings for new car models.

b. ABS/Combined Braking systems and daytime running lights for powered two-wheelers.

c. Automatic Emergency Braking Systems for cars.

d. Electronic stability control systems (ESC)

Such interventions can also be considered as a platform that practices the utilization of new processes in LMICs that can improve in the next stages

**Keywords:**

Vehicle Safety, Decade of Action for Road Safety, New Car Assessment Program

